# Replication Stress-Induced Chromosome Breakage Is Correlated with Replication Fork Progression and Is Preceded by Single-Stranded DNA Formation

**DOI:** 10.1534/g3.111.000554

**Published:** 2011-10-01

**Authors:** Wenyi Feng, Sara C. Di Rienzi, M. K. Raghuraman, Bonita J. Brewer

**Affiliations:** * Department of Genome Sciences, University of Washington, Seattle, Washington 98195; † Department of Biochemistry and Molecular Biology, Upstate Medical University, Syracuse, New York 13210

**Keywords:** chromosome fragile sites, double strand breaks, *mec1*, replication checkpoint, single-stranded DNA

## Abstract

Chromosome breakage as a result of replication stress has been hypothesized to be the direct consequence of defective replication fork progression, or “collapsed” replication forks. However, direct and genome-wide evidence that collapsed replication forks give rise to chromosome breakage is still lacking. Previously we showed that a yeast replication checkpoint mutant *mec1-1*, after transient exposure to replication impediment imposed by hydroxyurea (HU), failed to complete DNA replication, accumulated single-stranded DNA (ssDNA) at the replication forks, and fragmented its chromosomes. In this study, by following replication fork progression genome-wide via ssDNA detection and by direct mapping of chromosome breakage after HU exposure, we have tested the hypothesis that the chromosome breakage in *mec1* cells occurs at collapsed replication forks. We demonstrate that sites of chromosome breakage indeed correlate with replication fork locations. Moreover, ssDNA can be detected prior to chromosome breakage, suggesting that ssDNA accumulation is the common precursor to double strand breaks at collapsed replication forks.

Chromosome fragile sites (CFS) were first identified in humans as specific regions of constrictions, gaps, or breaks on metaphase chromosomes after cells were exposed to chemicals that inhibit DNA replication (Sutherland 1979). As CFSs are hot spots for genomic rearrangement, their identification is important for understanding the mechanisms of genomic instability induced by replication stress. Although the exact nature of molecular events causing chromosome fragility is still elusive, increasing evidence suggests that delayed or defective replication fork progression through fragile sites may be one of the underlying causes of chromosome fragility. Studies using the model organism *Saccharomyces cerevisiae* under a variety of conditions have also described chromosome fragility that stems either from intrinsic properties of DNA templates or from reagents that interfere with DNA replication and/or compromise the checkpoint control mechanism (Admire *et al.* 2006; Casper *et al.* 2009; Casper *et al.* 2008; Cha and Kleckner 2002; Lemoine *et al.* 2005; Raveendranathan *et al.* 2006). The unifying hypothesis for the observed chromosome fragility is that replication stress causes the destabilization or “collapse” of the replication forks at specific regions of the chromosomes where fragility occurs. The corollary to this hypothesis is that breakage at CFSs is correlated with altered replication fork progression under the stress conditions. However, direct evidence for a correlation between chromosome breakage and replication fork progression is still absent.

Previously, we reported that when checkpoint-deficient *mec1-1* cells are exposed to HU at the beginning of S phase, ssDNA accumulates at the replication forks that arise from origin activation (Feng *et al.* 2009). Moreover, after the removal of HU, replication forks are incapable of resuming DNA synthesis, and cells suffer extensive chromosome breakage. We hypothesized that chromosome breakage occurs, at least in part, as a consequence of chromosomes being under persistent tension exerted by the mitotic spindle. Consistent with this hypothesis was our observation that breakage was evident near a centromere located on a chromosome capable of bi-orientation on the spindle (*CEN2*) but not near a centromere on a chromosome unable to achieve bi-orientation (*CEN4*) (Feng *et al.* 2009). We also reasoned that it is unlikely that the chromosome breakage occurred as a direct consequence of the mechanical force exerted by the spindle; rather, chromosome breakage occurred as a result of a more open and vulnerable chromatin environment due to spindle extension. Thus, we believed that chromosome breakage might not be restricted to the centromere-proximal regions.

In this study, we set out to test the hypothesis that CFSs map to sites of replication fork “collapse” by simultaneously mapping replication fork progression and sites of chromosome fragility. We monitored replication fork progression indirectly by detecting ssDNA production at replication forks and identified chromosome breakage sites by direct mapping of double-stranded breaks (DSB) using a new method. As predicted, we saw a strong correlation between fork locations and sites of chromosome breakage. Furthermore, accumulation of ssDNA preceded the detectable appearance of DSBs, suggesting that ssDNA formation at replication forks is a precursor to chromosome breakage at those locations.

## Materials and Methods

### Yeast strains and media

HM14-3a (*MATa RAD53 bar1-1 his6 leu2-3,112 trp1-289*) and WFA34 (*MATa RAD53::rad53K227A(KanMX4) bar1-1 his6 leu2-3,112 trp1-289*) strains are derivatives of RM14-3a in an A364a background through gene conversions as previously described (Feng *et al.* 2006). BY2006 (*MATa mec1-1::HIS3 his3 leu2 trp1 ura3*), also an A364a derivative, was provided by Dr. Linda Breeden at the Fred Hutchinson Cancer Research Center. RCY378 (*MATa ho::LYS2 mec1::LEU2 lys2 ura3 leu2::hisG ade2::LK his4x arg4Ndel::mec1-4-kanMX4*) and the isogenic *MEC1* derivative RCY301 were provided by Dr. Rita Cha at the MRC National Institute for Medical Research. YSCL004 (*MATalpha ∆ho ∆hml::ADE1 ∆hmr::ADE1 ade1-110 leu2,3-112 lys5 trp1::hisG ura3-52 ade3::GAL10:HO Chr VI 97749 nt::HPH:HOcs Chr II 251662-251762 nt::HOcs:URA3*) was generously given by Jim Haber and C-S. Lee at Brandeis University. Cells were grown at 30° in synthetic complete medium unless otherwise indicated. Alpha factor was used at 200 nM for *bar1* strains and 3 μM for *BAR1* strains. Pronase was used at 25 μg/ml and 300 μg/ml for *bar1* and *BAR1* strains, respectively, to remove alpha factor from the culture medium. HU was added at 200 mM.

### CHEF gel electrophoresis

CHEF gel analysis was performed as described previously (Van Brabant *et al.* 2001). Electrophoresis was conducted at 14° for 26 hr with a switch time ramped from 60 to 120 sec at 200 volts. Standard procedures were used to ethidium bromide stain and photograph the gel.

### In-gel ssDNA labeling

Approximately 5 × 10^8^ cells were collected for each sample during the time course of HU exposure and subsequent recovery. The cells were then embedded in agarose plugs and spheroplasted using the same procedures as in the CHEF gel electrophoresis analysis. The agarose plugs were pre-equilibrated in 10 mM Tris-HCl, pH 8.0, 0.1 mM EDTA (5 ml per plug) for 30 min, followed by equilibration in 5 ml of 50 mM Tris-HCl pH 6.8, 5 mM MgCl_2_, and 10 mM β-mercaptoethanol for 30 min at room temperature. For each sliver of agarose plug containing 10^8^ cells in approximately 50 μl, 50 μl of labeling mix [50 mM Tris-HCl (pH 6.8); 5 mM MgCl_2_; 10 mM β-mercaptoethanol; 0.24 mM of each of dATP, dCTP, and dGTP; 0.12 mM of dTTP; 0.12 mM Cy5 or Cy3-dUTP; 250 μg/ml random hexamers; and 150 units of Klenow (exonuclease deficient, New England Biolab)] were added atop the plug, and the plug was incubated at 37° in the dark for 2 hr. The embedded labeled DNA was then electroeluted from the agarose plug in Spectra/Por dialysis tubing (Spectrum) with a 12,000-14,000 MWCO in 0.5× TBE at 110 volts at room temperature in the dark for 3 hr. The eluted DNA was then sonicated using a BioRuptor (Diagenode) to reduce the average size to 500 bp and purified using the Qiagen PCR Cleanup Kit. The resulting DNAs from a control and an experimental sample, which were differentially labeled with Cy-dUTP, were mixed together and readied for microarray analysis.

### In-gel DSB labeling

Sample preparation prior to labeling was performed as described above for the in-gel ssDNA labeling. Restriction digestion of DNA in agarose plugs was performed as described previously (Feng *et al.* 2009). After the agarose plugs were pre-equilibrated in 10 mM Tris-HCl (pH 8.0) and 0.1 mM EDTA, they were equilibrated in 1× End-Repair buffer [Epicentre, 33 mM Tris-acetate (pH 7.8), 66 mM KAc, 10 mM MgAc_2_, and 5 mM dithiothreitol] at 5 ml/plug for 30 min at room temperature. For each sliver of agarose plug containing 10^8^ cells in approximately 50 μl, 50 μl of End-Repair labeling mix (1× End-Repair buffer; 1 mM ATP; 0.24 mM of each of dATP, dCTP, and dGTP; 0.12 mM of dTTP; 0.12 mM Cy5 or Cy3-dUTP; and 3 μl of End-Repair enzyme mix) were added, and the plug was incubated at room temperature in the dark for 1 hr. Electroelution and subsequent analyses were performed identically as described above.

### Microarray analysis

The experimental and control DNA samples were mixed for cohybridization to the Agilent G4493A yeast 4x44K ChIP to chip DNA microarrays according to the manufacturer’s recommendations. Data extraction was performed using Agilent’s Feature Extraction software. After removing those array spots flagged by the software as anomalous, the ratio of background-subtracted fluorescent signals from the experimental to the control sample was calculated for each probe. The resulting ratios for all the probe locations on each chromosome were normalized to the total amount of signals in each fluorescent channel and smoothed with a 6 kb window using a Lowess smoothing algorithm as previously described (Feng *et al.* 2009). The “chromosome breakage profile” was generated by plotting the smoothed ratios against chromosome coordinates. Identification of peaks in the profiles was performed as previously described (Feng *et al.* 2009). The ssDNA and chromosome breakage mapping in *mec1* cells were each performed three times and twice, respectively, with reproducible profiles. The *Bam*HI, *Fsp*I, and HO cleavage experiments were performed only once. The significant chromosome breakage sites in *mec1* cells after 1 h recovery from exposure to HU obtained from two independent experiments and origin locations used for statistical tests are reported (supporting information, Table S1). All data files for raw ratios of ssDNA or breakage are available in File S1.

### Random simulation test

To test for an association between break sites and chromosomal features within 6 kb, the distance between each break site and its nearest chromosomal feature of interest was first established. Distances between breaks and chromosomal features were measured from midpoint to midpoint. Breaks with chromosomal features within 6 kb were counted. The null distribution of the number of breaks with a given chromosomal feature within 6 kb was determined by randomizing the location of break sites and determining the distance between these random breaks and the chromosomal features. Break sites were randomized by randomly selecting an equal number of positions on the microarray. Ten thousand simulations were run, and in each run, the number of random breaks with a given chromosomal feature within 6 kb was recorded. *P* values were obtained from the upper tail of the null distribution in which the number of breaks with a chromosomal feature within 6 kb was greater than or equal to that seen for the actual set of breaks. The list of Rad53-checked and unchecked origins used in these analyses were obtained from the previous study (Feng *et al.* 2006).

## Results

### Previously single-stranded regions of the genome remain single-stranded even after HU removal in *mec1* cells

**Figure 1:**
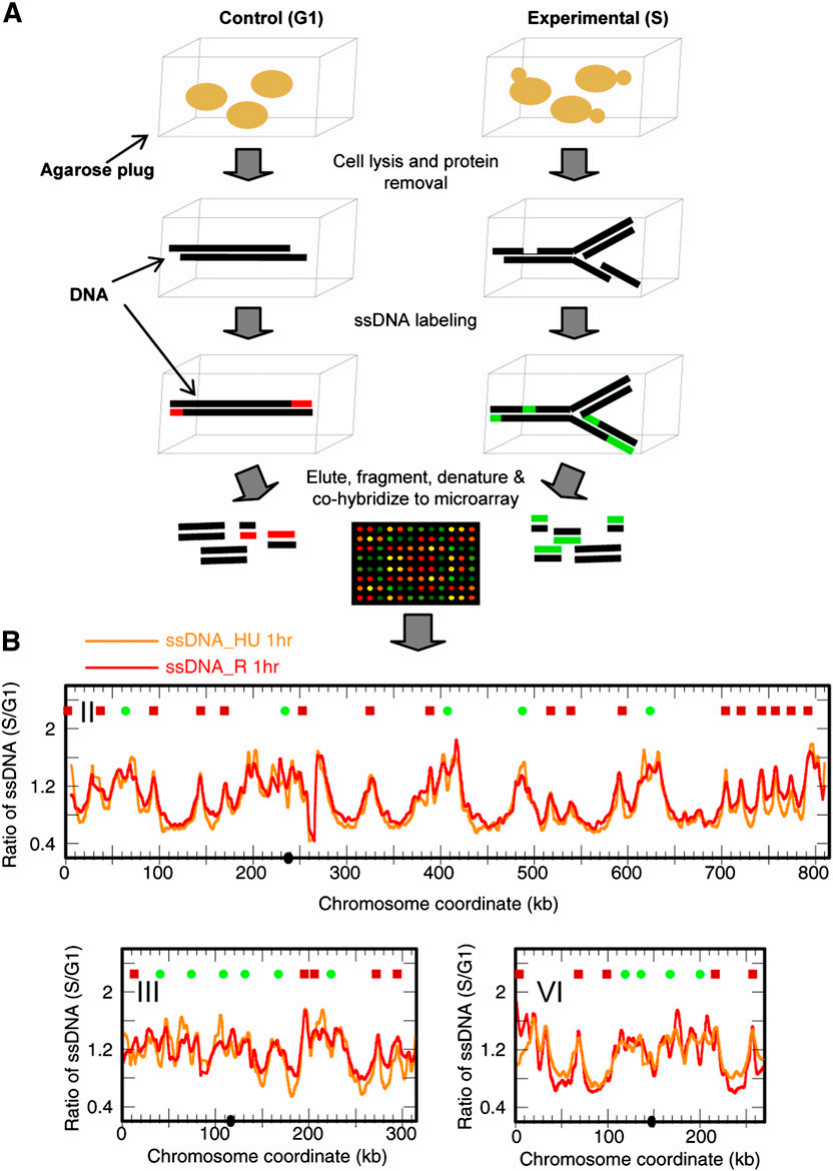
(A) Outline of experimental procedures for a modified in-gel ssDNA labeling and identification method. (B) ssDNA persists in *mec1* cells recovering from exposure to HU. The ssDNA profiles for *mec1-1* cells collected after cells were released from alpha factor arrest and exposed to 200 mM HU for 1 hr (“ssDNA_HU 1hr”; orange profile) and after recovering from 1 hr exposure in media without HU for another 1 hr (“ssDNA_R 1hr”; red profile) are shown. The symbols at the top of each graph indicate the locations of replication origins: red squares, checked (late/inefficient) origins and green dots, unchecked (early/efficient) origins (Feng *et al.* 2006). Chromosome numbers are indicated in Roman numerals. Centromere locations are shown as black dots on the X-axis. The same symbols are used for all remaining chromosome profiles throughout the article.

What regions of the *mec1* chromosomes are most susceptible to breakage in the presence of HU and during recovery from HU? Because we previously observed that extensive ssDNA formed at the replication forks in *mec1* cells exposed to HU and that the forks were incapable of resuming synthesis after HU removal, we wondered whether the ssDNA at the replication forks would persist and become sites of DSBs. We tested for persistence of ssDNA in *mec1* cells following a transient exposure to HU. We synchronized *mec1* cells in G1 with the mating pheromone alpha factor, followed by release into S phase in the presence of 200 mM HU, and then collected samples at the beginning (0 hr) and after 1 hr (HU 1hr). We then filtered the cell culture, allowed the cells to “recover” in fresh medium without HU, and collected samples after 1 hr (R 1hr). We have previously described a method based on random-primed labeling without denaturation of genomic template DNA to map ssDNA genome-wide (Feng *et al.* 2006). We have now modified this procedure (see *Materials and Methods*) and prepared genomic DNA by embedding cells in agarose plugs followed by spheroplasting (Figure 1A). We then performed ssDNA labeling in gel to minimize ssDNA production *in vitro*. An “S phase” sample, either HU 1hr or R 1hr, was paired with a control “G1 phase” sample, and the two samples were differentially labeled with Cy-conjugated dUTPs. Labeled DNA was eluted from the agarose, fragmented, and cohybridized to Agilent microarray slides. The relative amount of ssDNA in the “S phase” sample was calculated as the ratio of fluorescent signals from the “S phase” sample to that from the “G1 phase” sample for each chromosome coordinate. As shown in Figure 1B, ssDNA was detected near origins of replication in the HU 1hr sample (orange profile). The reproducibility of this new in-gel labeling methodology is exemplified by the correlation coefficients for the pair-wise comparisons of microarray data from three independent experiments: 0.91, 0.84, and 0.80. Interestingly, the ssDNA profiles of the R 1hr sample (red profile) and the HU 1hr sample were virtually identical (correlation coefficient = 0.93), indicating that the single-stranded gaps remained unfilled even after HU was removed. To validate this modified method, we also mapped ssDNA in a *rad53K227A* mutant in HU, which we had previously demonstrated, using the old methodology, to accumulate ssDNA at all known origins (Feng *et al.* 2006). The two ssDNA profiles were nearly identical with even better resolution attributed to the in-gel labeling method (Figure S1). We were also concerned that the ssDNA labeling we observed might be the result of exonuclease-deficient (exo–) Klenow’s strand displacement synthesis—extending the 3′-end of a nick in the DNA and displacing the downstream DNA strand—rather than the result of filling ssDNA gaps in the template. Therefore, we tested the labeling with Sequenase (an exo– T7 DNA polymerase that does not possess the strand displacement activity). The ssDNA profiles produced by Klenow and Sequenase are very similar, thus confirming that *in vitro* synthesis is labeling ssDNA gaps, not merely nicks, in *mec1* cells in HU (Figure S2).

### A new genome-wide chromosome breakage mapping method

If the ssDNAs that persist after removal of HU contribute to chromosome fragility, then their locations should correspond to sites of breakage. On a genome-wide level, meiotic DSBs have been mapped by two microarray-based methods (Blitzblau *et al.* 2007; Buhler *et al.* 2007; Cotta-Ramusino *et al.* 2005; Robine *et al.* 2007) and a whole-genome sequencing-based method (Pan *et al.* 2011). These methods monitor either Spo11 binding (via chromosome immunoprecipitation followed by microarray hybridization or sequencing, ChIP-chip, or ChIP-Seq) or the ssDNA tail left by Spo11 at the DSBs (via benzoyl-napthoyl-DEAE-cellulose ssDNA enrichment). CFSs can also be identified through the detection of phosphorylated γ-H2AX, which binds DSBs induced by a variety of conditions (Szilard *et al.* 2010). However, these methods do not label the terminal DSBs directly and could be prone to false positives. For instance, many Spo11 binding sites are not cleaved. Likewise, it is formally possible that with the ssDNA enrichment method, an internal ssDNA gap that is not associated with the DSB might be falsely identified as a product of DSB processing. It has also been reported that γ-H2AX foci were detected in the absence of apparent DSBs (Banath *et al.* 2004). Moreover, ChIP-chip or ChIP-Seq is not yet amenable to our study because it is unclear which proteins will be stably present at the HU-induced DSBs. Finally, reliably detecting DSBs is confounded by random breaks occurring *in vitro* during DNA isolation.

**Figure 2:**
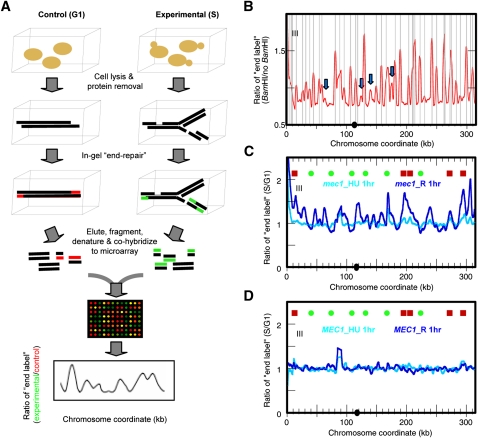
Chromosome breakage mapping. (A) Outline of microarray-based genome-wide chromosome breakage mapping. For details, see text. (B) End-labeled profile of Chr III from a control sample (log phase *mec1* cells) that contains *in vitro*–generated *Bam*HI ends. The gray vertical lines indicate positions of *Bam*HI digestion sites predicted from the sequence of the *S. cerevisiae* reference genome (S288C). Blue arrows indicate potential polymorphic *Bam*HI sites in the yeast strain used in this study. (C) End-labeled profile of *mec1* cells at “*mec1*_HU 1hr” (cyan) and “*mec1*_R 1hr” (blue). (D) End-labeled profile of *MEC1* cells at “*MEC1*_HU 1hr” (cyan) and “*MEC1*_R 1hr” (blue).

Therefore, we developed a new and versatile genome-wide DSBs mapping method (Figure 2A). To minimize DSBs generated by *in vitro* manipulations, we again prepared genomic DNA from yeast cells that were embedded in agarose plugs and processed for cell wall disruption and protein degradation. We then incorporated Cy-conjugated dUTP at DNA ends in gel with the End-It labeling kit (Epicentre), which utilizes both the polymerase and the 3′-5′ exonuclease activities of the T4 DNA polymerase and can process DNA with either 3′ or 5′ overhangs to generate primarily blunt ends. (The End-It kit also contains polynucleotide kinase, which adds a 5′-phosphate on the DNA end to facilitate cloning. This feature was not required for our experimental purpose, but it produced no ill effect.) We labeled DNA derived from an equal number of cells from the experimental sample (*e.g.*, R 1hr sample that contains DSBs) and from a control sample (nonreplicating DNA without apparent breaks) differentially with Cy3- and Cy5-conjugated dUTPs. We then eluted the DNA from agarose plugs and, after sonicating the DNA to reduce the average fragment size, cohybridized them to Agilent DNA microarrays. The relative level of DSBs was expressed as the ratio of fluorescence signals from the experimental sample to those from the control. Data smoothing and peak identification were performed as previously described (Feng *et al.* 2009).

We first performed a control experiment to map chromosome breakage in samples containing known DSBs. We prepared DNA embedded in agarose plugs as described above from exponentially growing *mec1* cells and digested the DNA in gel with the restriction enzyme *Bam*HI. We then differentially labeled the digested and the undigested control DNA in gel with Cy-conjugated dUTPs and carried out the procedures described above to obtain a “chromosome breakage profile” of the cells containing *Bam*HI ends (Figure 2B). The mapped DSBs correlated nearly perfectly with the known *Bam*HI sites in the genome. There were some sites mapped as chromosome breaks that did not correspond to known *Bam*HI sites (Figure 2B, blue arrows), suggesting that they might be polymorphisms in our laboratory strain A364a compared with the sequenced strain S288C. Similar results were obtained when we performed the same experiment using the blunt cutter *Fsp*I (Figure S3). However, the end-repair labeling of blunt *Fsp*I-digested DNA ends was not as efficient as it was for the sticky ends generated by *Bam*HI. We reasoned that the DSBs generated during the HU challenge are unlikely to be blunt ended and that this is a potential caveat of the method.

We also asked whether our method was able to detect *in vivo* DSBs induced by the HO endonuclease in YSCL004, a strain that contains three irreparable HO cut sites due to the lack of donor sequence (one site each on Chr II, III, and VI). The HO gene was controlled by the galactose-inducible Gal1 promoter. Log phase cells grown in media containing glycerol were split into two cultures. Glucose (Glu) or galactose (Gal) was added to the cultures to repress or induce HO activity, respectively. Both cultures were incubated for 1.5 hr to allow the nucleolytic events to proceed in the Gal culture before the addition of glucose to stop further HO cutting. DNA samples were prepared by spheroplasting after cells were embedded in agarose as described above, differentially end-labeled with Cy-dyes, and cohybridized to the microarray. The relative amount of DSBs in the Gal sample was quantified as the ratio of fluorescent signal from the Gal sample to that from the Glu sample. We detected all three HO cut sites as regions that are enriched for end-labeled signals in the Gal sample (Figure S4).

Finally, we performed another control experiment to assess how specific the end-repair reaction is for the DNA ends *vs.* internal stretches of ssDNA that are not associated with DSBs. Our previous observations indicated that chromosomes did not break in *mec1* cells during HU exposure but that they did contain extensive ssDNA (Figure 1B). If an internal ssDNA stretch were to contain an adjacent free 3′-OH group, it would be a suitable primer for the T4 DNA polymerase and skew the results of breakage mapping. As shown in Figure 2C (light-blue profile), the HU 1hr sample exhibited a rather flat breakage profile and did not show significant signals at the ssDNA regions near the origins. This observation suggests that the end-repair reaction does not label internal stretches of ssDNA efficiently and/or that the ssDNA gaps in the HU-treated *mec1* cells do not contain free 3′-OH groups.

### Chromosome breakage occurs at ssDNA locations

We mapped DSBs in the *mec1* sample that contained extensive chromosome breakage (R 1hr) shown previously (Feng *et al.* 2009), and we found specific labeling at multiple sites in the genome (Figure 2C, dark-blue profile). The magnitude of breakage in *mec1* cells was comparable to that at the *Bam*HI sites, but it was much lower than at the HO sites, demonstrating that chromosome breakage in the *mec1* cells was occurring genome-wide rather than at discrete sites (Feng *et al.* 2009). To confirm our results, we validated a chromosome breakage site on Chr II at 620 kb by indirect end-labeling (Figure S5). In contrast, the wild type (WT) cells in both the HU 1hr and the R 1hr samples did not exhibit significant levels of chromosome breakage (Figure 2D).

At first glance, the sites of breakage in *mec1* cells recovering from HU appear to occur at specific locations with no obvious correlation to known origins or other landmarks. However, when origins were parsed into the categories of “early/efficient” (origins that fire in HU in the presence of an activated checkpoint; unchecked origins) or “late/inefficient” (origins that are delayed in firing after activation of the checkpoint; checked origins), breakage sites were better correlated with forks that initiated from the checked origins (Figure 2C, red squares) than from the unchecked origins (Figure 2C, green dots). To perform this comparison, we first identified those chromosome positions where significant (above median level) breakage was observed. We then asked what percentage of these chromosome positions are within 6 kb of the nearest origin of replication. We chose a 6 kb cutoff because the Lowess smoothing window size of our microarray data was set at 6 kb. A significantly greater percentage of breakage sites was found near the checked origins (41.6%) than near the unchecked origins (11.7%) (*P* < 10^−15^) in a two-sample test for equality of proportions with continuity correction. To test this correlation more rigorously, we also performed a random simulation test and found that the breakage sites were correlated with the checked origins (*P* < 0.0001) but not with the unchecked origins (*P* = 0.1851) within a 6 kb distance. We interpret these results as follows. In the *mec1* mutant, all origins fire (Feng *et al.* 2009); however, it appears that the temporal pattern of origin activation remains, as the ssDNA profiles indicated that replication forks migrated away from the unchecked (early) origins but were still in the vicinity of the checked (late) origins. This result suggests that chromosome breakage is correlated with replication fork progression.

**Figure 3:**
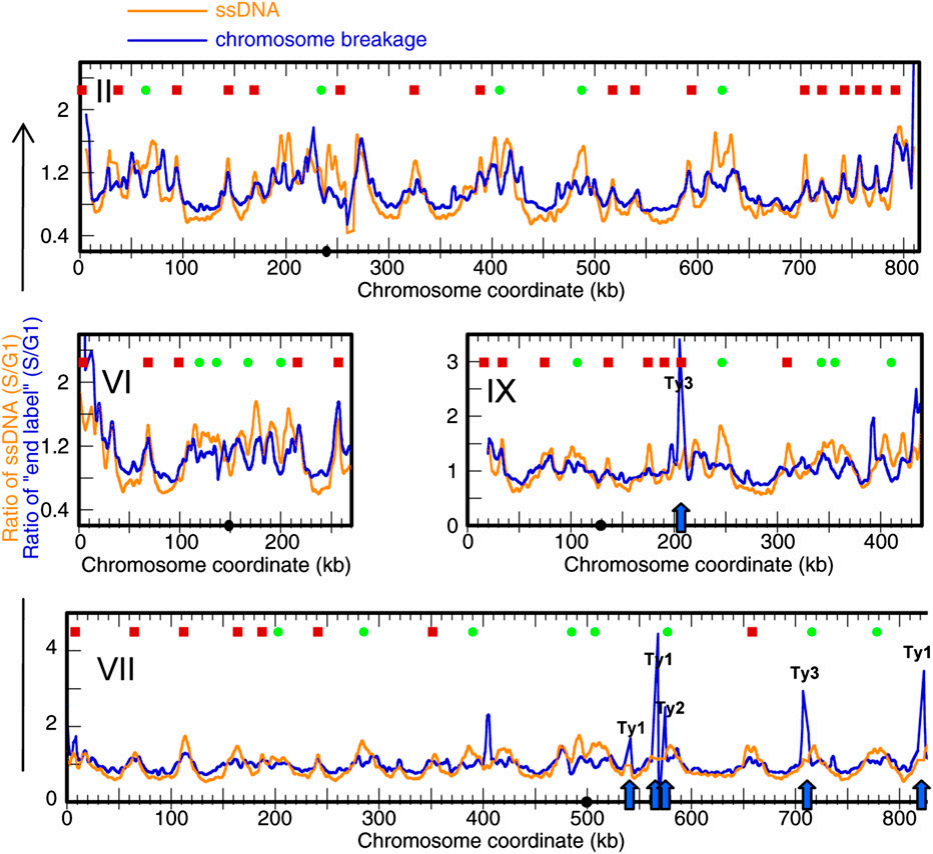
Correlation of sites of breakage with ssDNA formation. The chromosome breakage profile of sample “R 1 hr” (blue) resembles the ssDNA profile detected in sample “HU 1 hr” (orange). Profiles for Chr II, VI, VII (left) and IX (right) are shown. The positions of Ty elements are indicated by blue arrows; the category of Ty element is indicated by a label above each peak.

Comparison of the breakage profile of the R 1hr sample and the ssDNA profile of the HU 1hr sample (which records the positions of replication forks) reveals significant similarity (correlation coefficient = 0.64; Figure 3). Cell synchrony was reduced for the R 1hr sample (2 hr after release from G1 arrest), which likely contributed to the relatively lower resolution of signals, as two independent breakage profiles of the R 1hr samples showed a correlation coefficient of 0.67. That the regional accumulation of ssDNA in HU occurs before breaks are detected in the recovery phase supports the hypothesis that ssDNA formation precedes the occurrence of chromosome breakage and dictates their location. Thus, the identification of ssDNA in the genome can be used to predict sites of chromosome breakage, an observation that has profound implications for the study of CFSs in the human genome. The end-labeling method also detected strong signals at Ty elements (Figure 3, Chr VII and IX, arrows). We entertained the possibility that chromosome breakage is generated at these genomic locations as replication forks progress through the Ty elements, but we found no evidence of breakage at these chromosomal sites by indirect end-labeling of Southern blots (data not shown). Given that Ty3 transposition is cell cycle regulated (Menees and Sandmeyer 1994), we speculate that we may be detecting double-stranded DNA templates arising from cDNA synthesis and replication of Ty elements. Because Ty elements are repetitive, we could not determine with certainty where they map to in the genome using the array-based method.

### Chromosome breakage is correlated with replication fork progression

**Figure 4:**
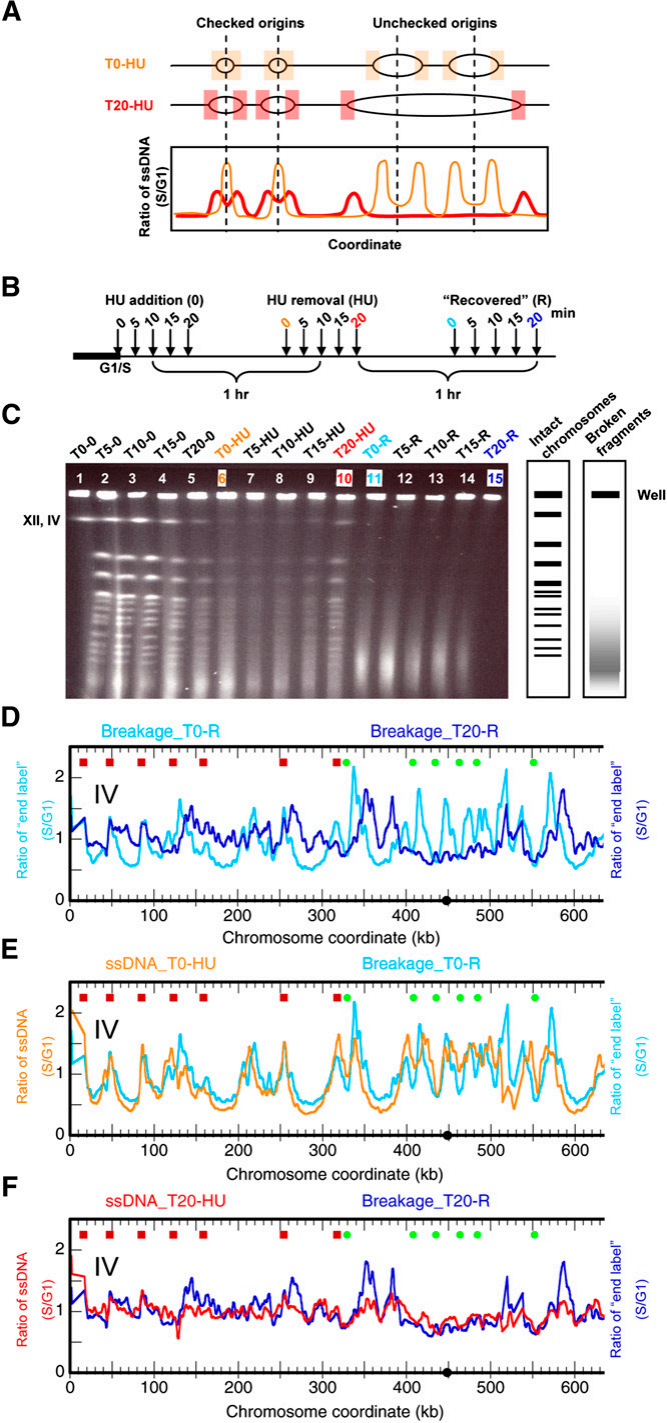
Chromosome breakage is correlated with replication fork progression. (A) Schematic representation of the scenarios of replication fork progression and the resulting ssDNA profiles in samples that were released into S phase in the presence of HU at different times. The nomenclature of samples is described in the main text. (B) Experimental scheme: *mec1* cells were released from alpha factor arrest into medium containing HU at the indicated times. The cells were exposed to HU for 1 hr followed by recovery in fresh medium without HU for 1 hr. For details, see text. (C) CHEF gel electrophoresis of samples from panel B. Chromosome breakage is evident in the recovery “R” samples as indicated. The position of Chr IV, which comigrates with Chr XII, is indicated. (D) Chr IV breakage profiles of recovery “R” samples from cells released into HU at the beginning of the G1/S transition (T0-R; cyan) or after an elapsed 20 min (T20-R; blue). (E) Chr IV breakage profile of the T0-R sample in panel D (cyan) overlaid with ssDNA profile of the cells before recovery (T0-HU; orange). (F) Chr IV breakage profile of the T20-R sample in panel D (blue) overlaid with ssDNA profile of the cells before recovery (T20-HU; red). (D–F) The left portion of Chr IV is shown in each profile.

We have demonstrated that the locations of chromosome breakage are correlated with ssDNA production at stalled replication forks, providing the first direct evidence that collapsed replication forks lead to DSBs. However, as many of the collapsed forks in HU occur near checked (late/inefficient) origins of replication, it is formally possible that this correlation of chromosome breakage with forks is unique to this specific subset of origins of replication rather than to the process of fork progression itself. To subject our hypothesis to a more rigorous test, we allowed replication forks to travel greater distances from the origins before HU treatment and then measured the sites of chromosome breakage in samples recovering from HU (Figure 4A). If it were the ssDNA at forks that contributed to chromosome fragility rather than late/inefficient origins *per se*, then the break sites should now be found at varying distances from these origins.

The experimental scheme is illustrated in Figure 4B. After synchronizing *mec1* cells at the G1/S boundary with alpha factor, we split the cell culture into five aliquots. We released the cells into S phase by pronase addition and added 200 mM HU at 0, 5, 10, 15, and 20 min after the release to the five aliquots denoted as T0, T5, T10, T15, and T20, respectively. We exposed each cell culture to HU for 1 hr before collecting the samples. We then washed the cells and transferred them into fresh medium without HU to let them “recover” for 1 hr. For clarity, we refer to the samples by their aliquot number followed by the description of the treatment they received: “T#” refers to the elapsed time (in minutes) after alpha factor removal and before HU addition; “HU” for a 1 hr exposure to HU; and “R” for a 1 hr recovery from HU. Thus, T5-HU designates the sample in which HU was added at 5 min after the release to S phase and which had been exposed to HU for 1 hr. Likewise, T20-R designates the sample in which HU was added at 20 min after release to the S phase, incubated for 1 hr in HU, and then allowed to recover for 1 hr after HU was removed.

DNA was prepared from each sample and subjected to contour-clamped homogeneous electric field (CHEF) gel electrophoresis as previously described (Feng *et al.* 2009). As cells enter S phase, linear chromosomes became depleted from the gel as the branched replicating chromosomes were trapped in the well (most obviously seen for the large chromosomes in T20; Figure 4C, lane 5). This property was even more striking for the HU-treated samples (lanes 6-10) where replication forks were slowed. The reappearance of the large chromosomes in the T20-HU sample (lane 10) suggests that many cells were able to complete replication when HU was added at this late time in S phase. However, regardless of when the cells encountered HU, they all exhibited chromosome breakage during recovery (lanes 11-15). We then tested the correlation between fork progression and chromosome breakage: if forks had proceeded away from the origins, we would expect the sites of ssDNA to reflect this fork migration and, more importantly, the breakage sites to be different. We mapped chromosome breakage in the T20-R sample in which replication forks migrated the farthest, and then we mapped chromosome breakage in the control sample T0-R. As shown in Figure 4D, the two breakage profiles were indeed different (correlation coefficient = 0.17). Chromosome breakage at early replicating regions was reduced (Figure 4D, coordinates 400-500 kb) and more broadly distributed in the regions replicated by forks from unchecked origins.

To quantify the changes in breakage patterns, we again identified those chromosome positions where significant (above median level) breakage was observed. We then asked whether the break sites were correlated with origin locations within a 6 kb distance in the random simulation test. As was consistent with previous observations, neither the T0-R nor the T20-R sample showed correlation between the breakage sites and the unchecked origins (Table 1). As before, the break sites in the T0-R sample were correlated with checked origins (*P* = 0.0032); however, the break sites in the T20-R sample were not (*P* = 0.8276; Table 1). These observations indicate that chromosome breakage is less well correlated with origin locations when replication forks have sufficient nucleotides and time to move into flanking regions.

**Table 1:** Correlation between origins of replication and chromosome breakage sites

Chromosomal feature	T0-R break sites (639)		T20-R break sites (788)	
	No. of break sites found near chromosomal feature	*P* value of simulation test	No. of break sites found near chromosomal feature*^a^*	*P* value of simulation test
Unchecked origins (105)	25	1	39	1
Checked origins (210)	228	0.0032	231	0.8276

Results of the random simulation tests for the correlation between chromosome breakage sites and various chromosome features within a 6 kb distance.

a The numbers of break sites and other chromosome features are shown in brackets. The numbers of break sites found near most chromosomal features in the T20-R sample are greater than those in the T0-R sample because the total number of significant (above median level) break sites in the T20-R sample is greater than in the T0-R sample (788 *vs.* 639).

Finally, we also monitored replication fork progression by ssDNA mapping in the T0-HU and T20-HU samples. Our results once again showed that the ssDNA profiles of the samples in HU prior to the occurrence of breakage were very similar to the breakage profiles (Figure 4E, F). The correlation coefficient between the T0-HU ssDNA and T0-R breakage profiles was 0.74 and that between the T20-HU ssDNA and T20-R breakage profiles was 0.65. Note that there was a negative, albeit weak, correlation (coefficient = -0.25) between the T0-HU ssDNA and T20-R breakage profiles, suggesting that the origin-proximal regions were protected from chromosome breakage if the forks were allowed to migrate away from them before encountering HU. Thus, our analyses demonstrated that (1) chromosome breakage is correlated with replication fork progression; (2) ssDNA formation is detected prior to chromosome breakage; and (3) sites of ssDNA formation predict the sites of chromosome breakage.

### ssDNA is also detected prior to chromosome breakage in a *mec1^ts^* mutant without external replication stress

**Figure 5:**
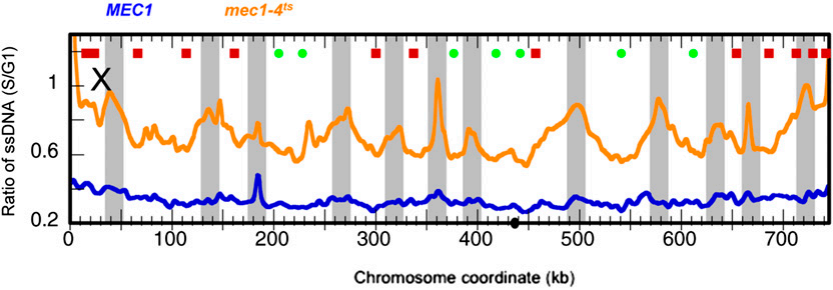
ssDNA predicts sites of eventual chromosome breakage in a *mec1-4^ts^* mutant. *MEC1* or *mec1-4^ts^* cells were released from alpha factor arrest to enter S phase at the restrictive temperature (37°), and samples were collected after 40 min. Plotted are ssDNA profiles of the *MEC1* control sample (blue) and the *mec1-4^ts^* sample (orange). The light-gray shaded boxes are replication termination regions that show elevated levels of ssDNA in the *mec1-4^ts^* sample.

On the basis of our results, we postulated that ssDNA formation as a result of replication stress is a common precursor to DSBs. We tested this hypothesis by asking whether we could detect ssDNA at other replication stress–induced chromosome breakage sites prior to breakage taking place. It was reported that a temperature-sensitive *mec1-4* mutant has defective replication fork progression at the restrictive temperature, without external challenge by HU, and experiences chromosome breakage later in the cell cycle (Cha and Kleckner 2002). The breakage occurred at regions of the genome described as “replication slow zones” (RSZ), which are primarily replication termination sites. We set out to determine whether and when ssDNA formation is detected in the RSZs. We mapped ssDNA in *mec1-4* as well as *MEC1* cells in mid–S phase (40 min after release from the G1 arrest). As shown in Figure 5, *MEC1* cells showed a background level of ssDNA, indicating that replication forks were not stalled at specific regions of the genome. In contrast, the *mec1-4* cells showed distinct patterns of elevated levels of ssDNA near the replication termini, the very regions where chromosome breakage was shown to take place (Cha and Kleckner 2002). Although chromosome breakage was not observed until 4 to 6 hr after cells were released from G1, ssDNA was observed after only 40 min.

## Discussion

We have developed a genome-wide chromosome breakage mapping method and used it to identify chromosome breakage in checkpoint-deficient *mec1-1* cells after exposure to and recovery from HU. It is a versatile method that can be applied to any system in which persistent DSBs are generated. We demonstrated that chromosome breakage is correlated with replication fork progression, providing direct evidence that chromosome fragility results from destabilization of the replication forks in the absence of the checkpoint when the nucleotide pool is reduced/depleted. Our study also demonstrated that prior to the occurrence of chromosome breakage, ssDNA could be detected at the sites of breakage. We thus hypothesized that ssDNA formation is a common precursor to chromosome fragility during replication stress. Using a *mec1-4* temperature-sensitive allele that exhibits chromosome fragility without external replication stress, we showed that ssDNA was indeed detectable at the sites of eventual chromosome breakage, lending additional support to our hypothesis. Our data provide a conceptual model where different forms of replication stress, despite having presumably different targets at the replication fork, cause cells to generate ssDNA at the fork as a consequence of either uncoupled synthesis on the two DNA templates or nucleolytic processing of synthesized DNA. We are currently determining the mechanisms of ssDNA formation during different replication stresses. We suggest that the detection of ssDNA can be used as a tool to identify sites of chromosome fragility and may signal when particular replication forks encounter impediments and/or become unstable.

## Supplementary Material

Supporting Information
