# Supplementary material for: Replication Stress-Induced Chromosome Breakage Is Correlated with Replication Fork Progression and Is Preceded by Single-Stranded DNA Formation
Source: G3 (Bethesda). 2011 Oct 1;1(5):327–35. doi: 10.1534/g3.111.000554 (PMC3276152; doi:10.1534/g3.111.000554)
Supplement: Supporting Information [file supp_1_5_327__index.html]

Supporting Information 

# Replication Stress-Induced Chromosome Breakage Is Correlated with Replication Fork Progression and Is Preceded by Single-Stranded DNA Formation

## Supporting Information for Feng *et al.*, 2011

**Files in this Data Supplement:**

- Supporting Information - Figures S1-S5, File S1, and Table S1 (PDF, 976 KB)
- Figure S1 - ssDNA profiles for Chr X of *rad53K227A* cells after exposure to 200 mM HU using the current in-gel labeling methodology (green) in comparison with the previously described DNA preparation and ssDNA labeling (orange) in Feng et al., 2006 (PDF, 132 KB)
- Figure S2 - Comparison between random-primed in-gel ssDNA labeling by Klenow (orange) and Sequenase (cyan) (PDF, 212 KB)
- Figure S3 - Comparison between breakage profiles for Chr II of cells containing *Bam*HI (top) or FspI-induced (bottom) DNA ends (PDF, 236 KB)
- Figure S4 - "End-repair" detected *in vivo* DSBs generated by the HO endonuclease (PDF, 216 KB)
- Figure S5 - Verification of chromosome breakage site on Chr II by indirect end-labeling as previously described 8 (PDF, 132 KB)
- File S1 - Data files for raw ratios of ssDNA or breakage (.zip, 4.4 MB)
- Table S1 - Significant chromosome breakage sites in *mec1* cells after one hour recovery from exposure to HU - obtained from two independent experiments, and the origin locations used for statistical tests (.txt, 28 KB)
