# Supplementary material for: Replication Stress-Induced Chromosome Breakage Is Correlated with Replication Fork Progression and Is Preceded by Single-Stranded DNA Formation
Source: G3 (Bethesda). 2011 Oct 1;1(5):327–35. doi: 10.1534/g3.111.000554 (PMC3276152; doi:10.1534/g3.111.000554)
Supplement: Supporting Information [file supp_1.5.327_000554.pdf]

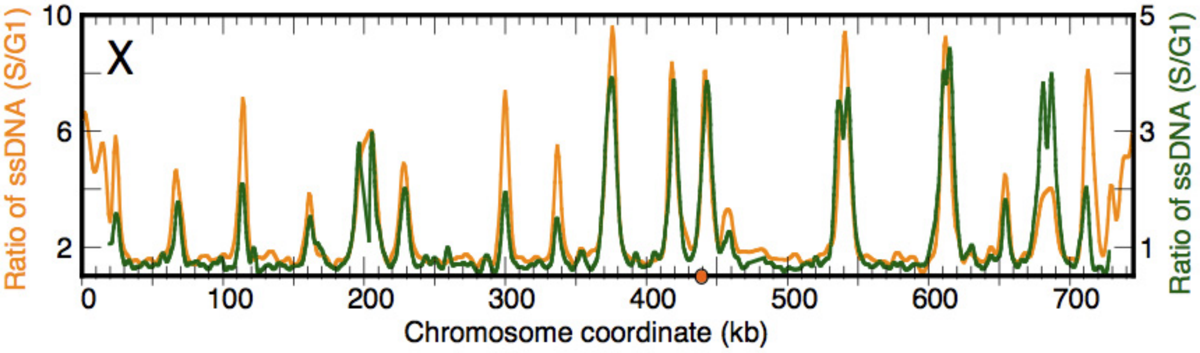

Figure S1 ssDNA profiles for Chr X of *rad53K227A* cells after exposure to 200 mM HU using the current in-gel labeling methodology (green) in comparison with the previously described DNA preparation and ssDNA labeling (orange) in Feng et al., 2006. Log phase *rad53* cells were synchronized with α factor and released into S phase in the presence of 200 mM HU for 1 hr. Cells collected from a G1 control sample as well as the S phase sample (HU 1hr) were collected and embedded in agarose, followed by spheroplasting, in-gel random-primed labeling by Klenow, differentially with Cy dyes, electroelution and co-hybridization to microarray as described in Methods. Ratios of ssDNA from the published data were plotted on the left Y axis and those from the current experiment were plotted on the right Y axis. The orange dot on the X axis denotes the centromere.

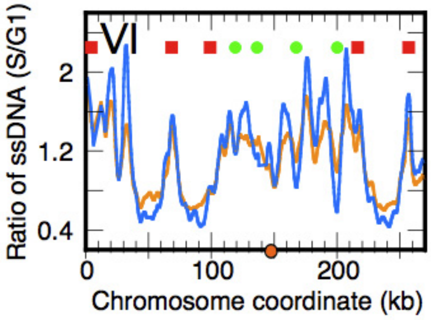

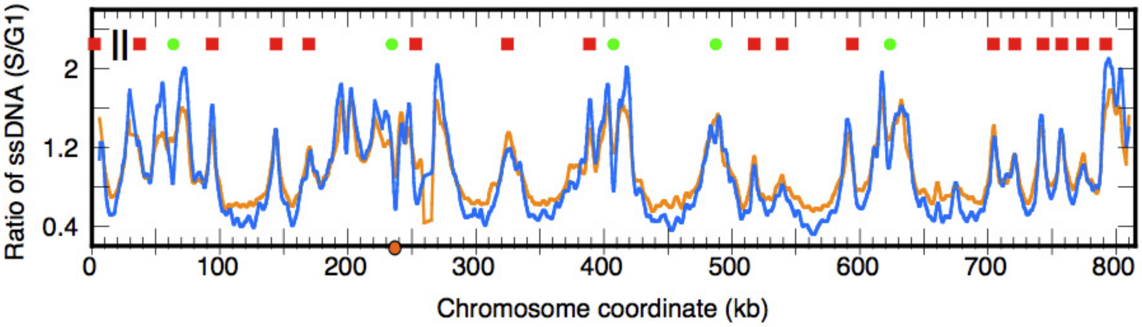

Figure S2 Comparison between random-primed in-gel ssDNA labeling by Klenow (orange) and Sequenase (cyan). *mec1* cells exposed to HU for 1 hr after synchronous release from α factor arrest were collected and embedded in agarose followed by spheroplasting as described in Methods. Labeling by Klenow was performed as described in Fig. 1. Labeling by Sequenase was performed similarly but with the following reaction mix: 40 mM Tris-HCl, pH 7.5; 10 mM $MgCl_2$; 5 mM dithiothreitol; 300 µM each of dATP, dCTP and dGTP; 150 µM dTTP; 150 µM Cy5 or Cy3-dUTP; 80 µM random hexamers, 30 units Sequenase (Agilent). The labeling reaction by Sequenase was incubated at 25°C for 1 hr before electroelution and subsequent analyses which were performed identically to samples labeled by Klenow. The orange dots on the X axes denote the centromere. Rad53- checked (red squares) and unchecked (green dots) origins are indicated on top the graphs.

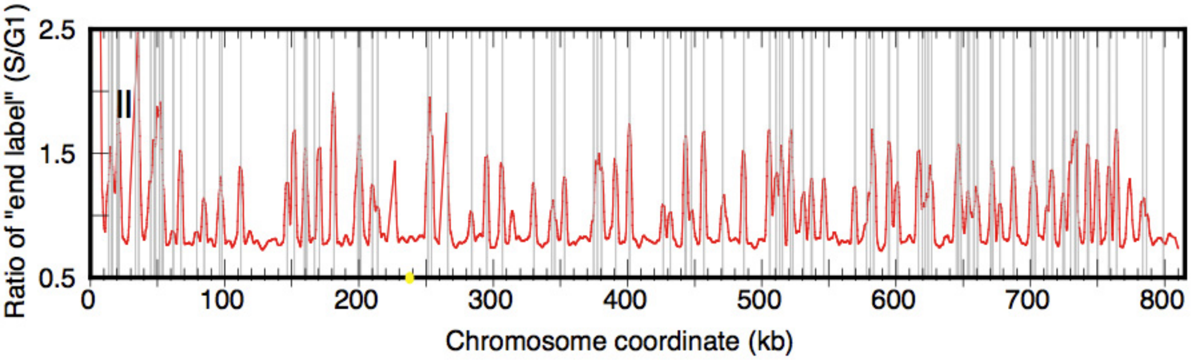

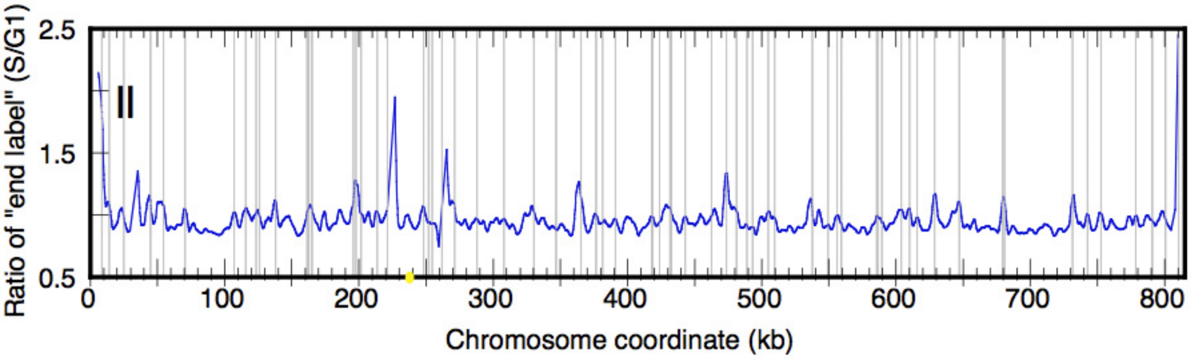

Figure S3 Comparison between breakage profiles for Chr II of cells containing *Bam*HI (top) or FspI-induced (bottom) DNA ends. The yellow dots denote the centromere. Grey lines indicate positions of known restriction sites for *Bam*HI (top) or *Fsp*I (bottom).

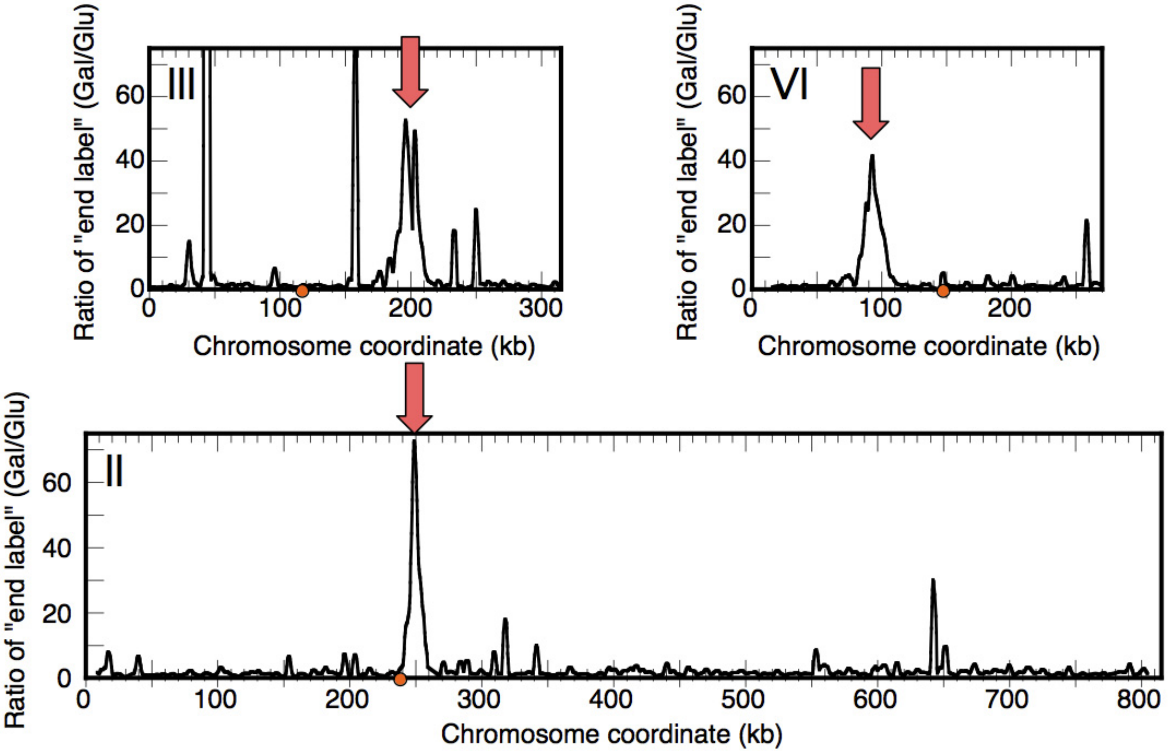

Figure S4 "End-repair" detected *in vivo* DSBs generated by the HO endonuclease. YCSL004 cells were grown to 5 x 10^6 cells/ml in YEP media supplemented with 3% glycerol at 30°C. The culture was split into two aliquots and glucose (Glu) or galactose (Gal) was added at 2% to the two aliquots respectively. The cultures were incubated at 30°C for 1.5 hrs before glucose was added to the Gal culture at 2% to stop further digestion by the HO nuclease. Samples were collected from these cultures and embedded in agarose as described in Methods, followed by spheroplasting and subsequent microarray labeling and detection. Shown are the ratios of "end label" of Gal/Glu samples after Lowess smoothing or the chromosome breakage profile for Chr II, III and VI. The positions of the HO cut sites are indicated by arrows. Note that the spurious peaks on Chr III at 42, 106, 232 and 250 kb as well as the majority of spurious peaks on Chr II and VI are all due to a singleton outlier probe on the microarray. In contrast, all the chromosome breakage peaks at the HO cut sites are comprised of approximately 100 consecutive probes on the microarray.

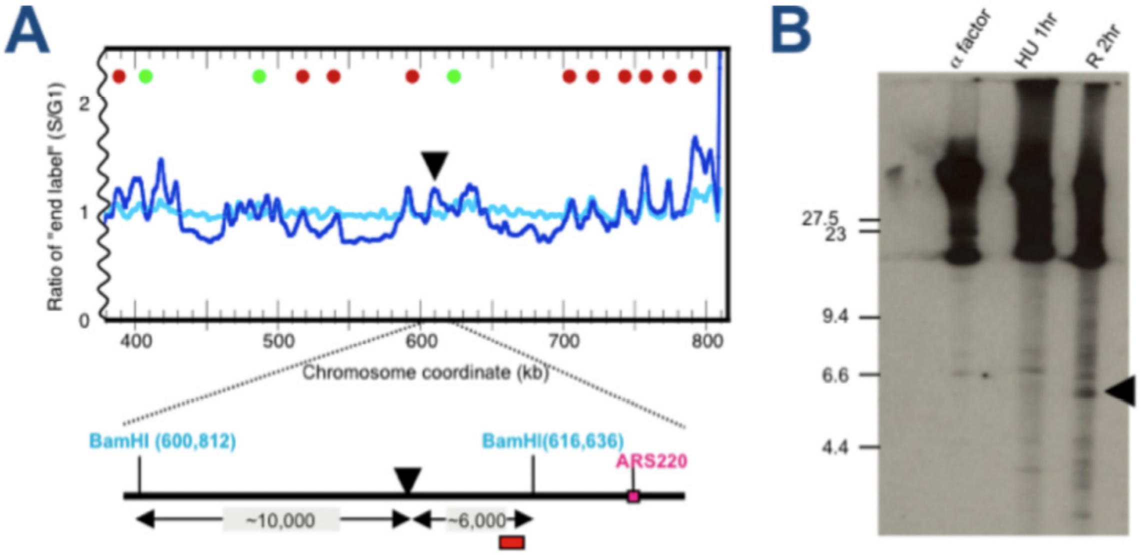

Figure S5 Verification of chromosome breakage site on Chr II by indirect end-labeling as previously described [8]. (A) Top: Chromosome breakage profile for the right half of Chr II for the HU 1hr (cyan) and the R 1hr (dark blue) samples. The breakage site chosen for analysis is indicated by an arrowhead. The Rad53-checked (red) and unchecked origins (green) are indicated as dots on top of the graph. Bottom: Structure of the *Bam*HI fragment harboring the break site that is analyzed by indirect end-labeling. The position of the Southern probe is indicated by a red bar. (B) Chromosomal DNA from *mec1* cells from the G1 control ("α factor"), after 1 hr exposure to HU ("HU 1hr") and recovering for 2 hr after HU exposure ("R 2 hr") were embedded in agarose and in-gel digested with *BamH*I before electrophoretic separation. Southern blotting was performed with the probe indicated in (A). The predicted fragment in the "R 2 hr" sample as a result of the breakage is indicated. Molecular weight markers in "kb" are shown on the left.

File S1

Data files for raw ratios of ssDNA or breakage

File S1 is available for download as a compressed text file at

http://www.g3journal.org/lookup/suppl/doi:10.1534/g3.111.000554/-/DC1/FileS1.zip.

Table S1

Significant chromosome breakage sites in *mec1* cells after one hour recovery from exposure to HU - obtained from two independent experiments, and the origin locations used for statistical tests.

Table S1 is available for download as a text file at http://www.g3journal.org/lookup/suppl/doi:10.1534/g3.111.000554/-/DC1/TableS1.txt.
