# Supplementary material for: Replication Stress-Induced Chromosome Breakage Is Correlated with Replication Fork Progression and Is Preceded by Single-Stranded DNA Formation
Source: G3 (Bethesda). 2011 Oct 1;1(5):327–35. doi: 10.1534/g3.111.000554 (PMC3276152; doi:10.1534/g3.111.000554)
Supplement: Supporting Information [file supp_1.5.327_FigureS1.pdf]

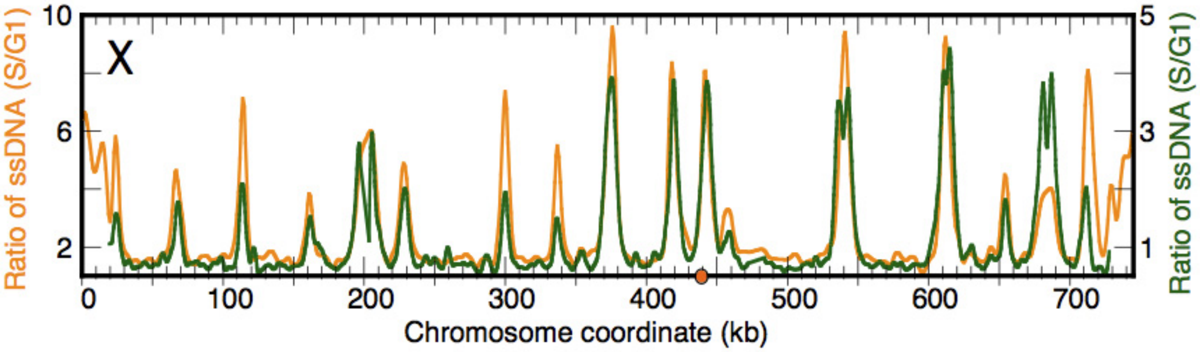

Figure S1 ssDNA profiles for Chr X of *rad53K227A* cells after exposure to 200 mM HU using the current in-gel labeling methodology (green) in comparison with the previously described DNA preparation and ssDNA labeling (orange) in Feng et al., 2006. Log phase *rad53* cells were synchronized with α factor and released into S phase in the presence of 200 mM HU for 1 hr. Cells collected from a G1 control sample as well as the S phase sample (HU 1hr) were collected and embedded in agarose, followed by spheroplasting, in-gel random-primed labeling by Klenow, differentially with Cy dyes, electroelution and co-hybridization to microarray as described in Methods. Ratios of ssDNA from the published data were plotted on the left Y axis and those from the current experiment were plotted on the right Y axis. The orange dot on the X axis denotes the centromere.
