# Supplementary material for: Replication Stress-Induced Chromosome Breakage Is Correlated with Replication Fork Progression and Is Preceded by Single-Stranded DNA Formation
Source: G3 (Bethesda). 2011 Oct 1;1(5):327–35. doi: 10.1534/g3.111.000554 (PMC3276152; doi:10.1534/g3.111.000554)
Supplement: Supporting Information [file supp_1.5.327_FigureS2.pdf]

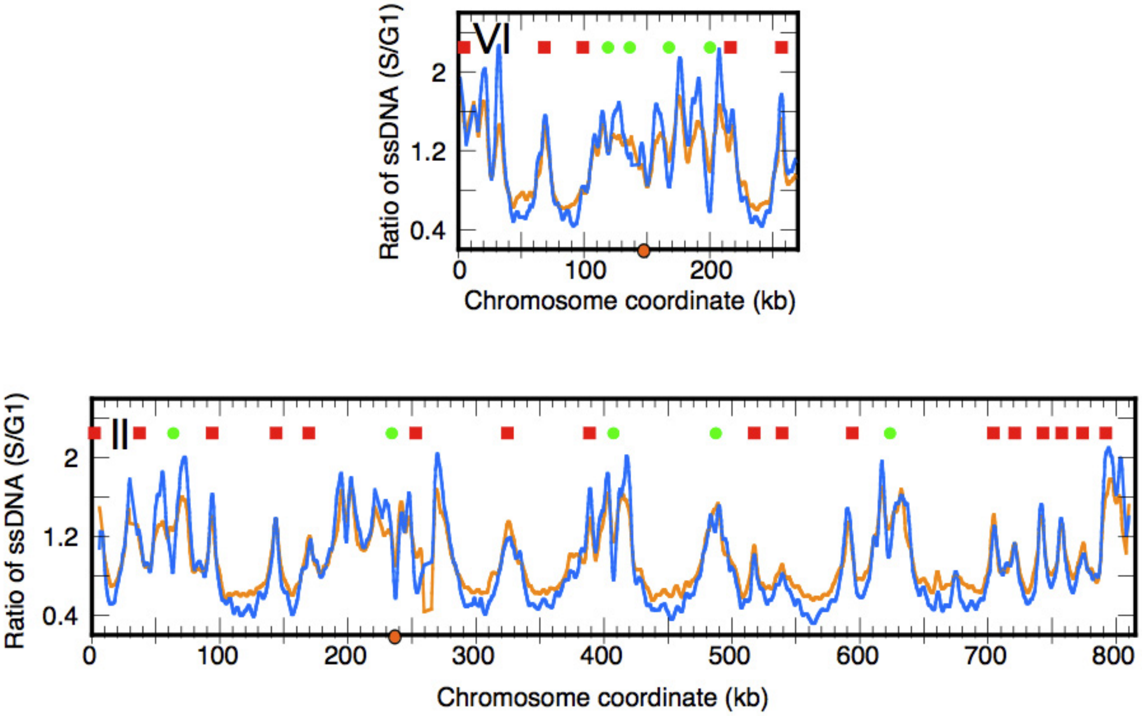

Figure S2 Comparison between random-primed in-gel ssDNA labeling by Klenow (orange) and Sequenase (cyan). *mec1* cells exposed to HU for 1 hr after synchronous release from α factor arrest were collected and embedded in agarose followed by spheroplasting as described in Methods. Labeling by Klenow was performed as described in Fig. 1. Labeling by Sequenase was performed similarly but with the following reaction mix: 40 mM Tris-HCl, pH 7.5; 10 mM $MgCl_2$; 5 mM dithiothreitol; 300 µM each of dATP, dCTP and dGTP; 150 µM dTTP; 150 µM Cy5 or Cy3-dUTP; 80 µM random hexamers, 30 units Sequenase (Agilent). The labeling reaction by Sequenase was incubated at 25°C for 1 hr before electroelution and subsequent analyses which were performed identically to samples labeled by Klenow. The orange dots on the X axes denote the centromere. Rad53- checked (red squares) and unchecked (green dots) origins are indicated on top the graphs.
