# Supplementary material for: Replication Stress-Induced Chromosome Breakage Is Correlated with Replication Fork Progression and Is Preceded by Single-Stranded DNA Formation
Source: G3 (Bethesda). 2011 Oct 1;1(5):327–35. doi: 10.1534/g3.111.000554 (PMC3276152; doi:10.1534/g3.111.000554)
Supplement: Supporting Information [file supp_1.5.327_FigureS3.pdf]

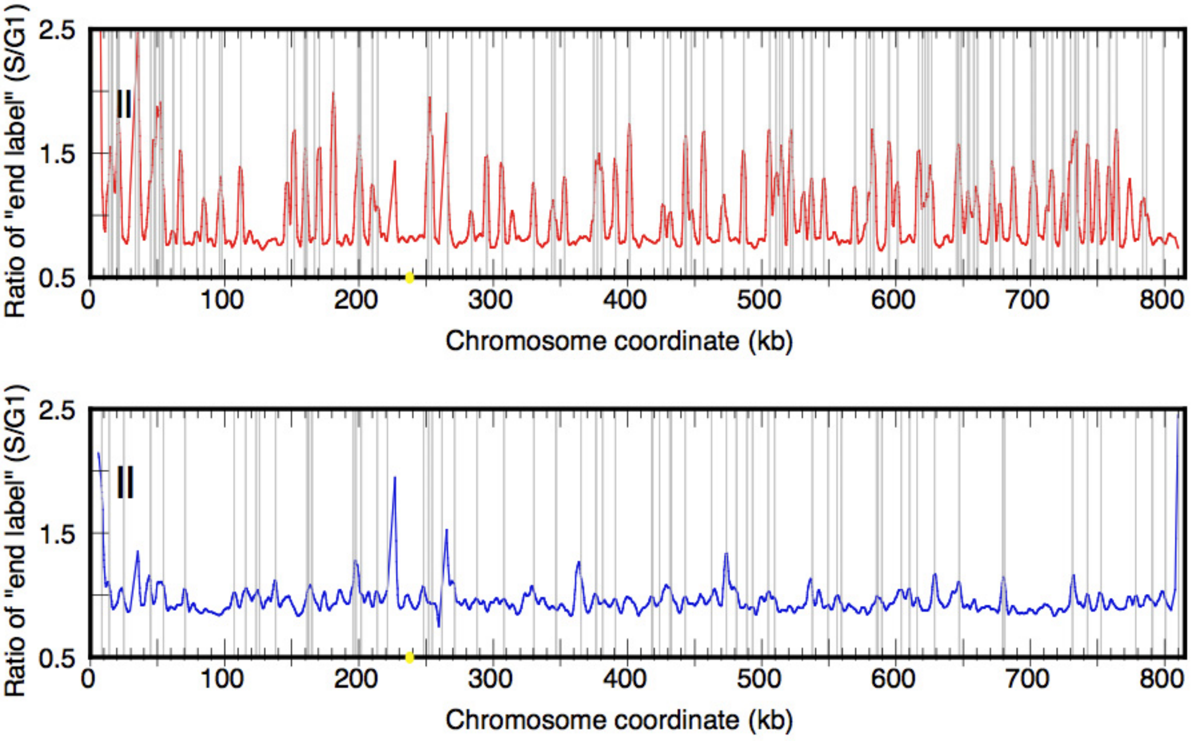

Figure S3 Comparison between breakage profiles for Chr II of cells containing *Bam*HI (top) or FspI-induced (bottom) DNA ends. The yellow dots denote the centromere. Grey lines indicate positions of known restriction sites for *Bam*HI (top) or *Fsp*I (bottom).
