# Supplementary material for: Replication Stress-Induced Chromosome Breakage Is Correlated with Replication Fork Progression and Is Preceded by Single-Stranded DNA Formation
Source: G3 (Bethesda). 2011 Oct 1;1(5):327–35. doi: 10.1534/g3.111.000554 (PMC3276152; doi:10.1534/g3.111.000554)
Supplement: Supporting Information [file supp_1.5.327_FigureS4.pdf]

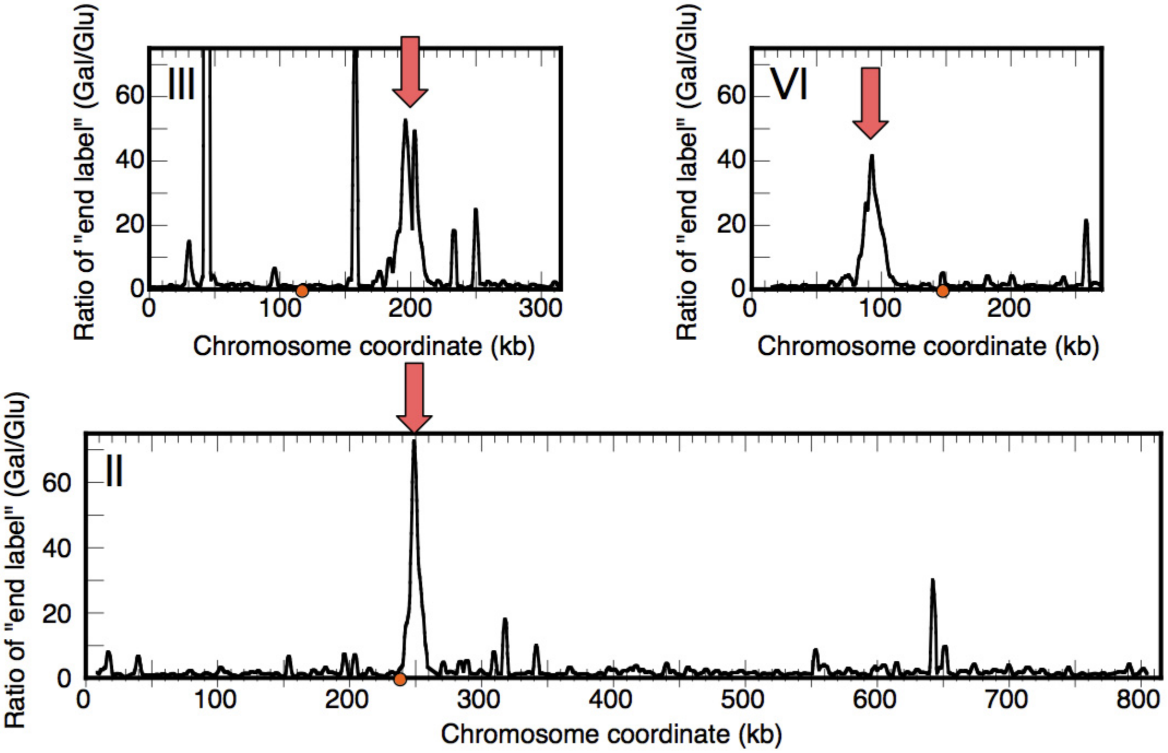

Figure S4 "End-repair" detected *in vivo* DSBs generated by the HO endonuclease. YCSL004 cells were grown to 5 x 10^6 cells/ml in YEP media supplemented with 3% glycerol at 30°C. The culture was split into two aliquots and glucose (Glu) or galactose (Gal) was added at 2% to the two aliquots respectively. The cultures were incubated at 30°C for 1.5 hrs before glucose was added to the Gal culture at 2% to stop further digestion by the HO nuclease. Samples were collected from these cultures and embedded in agarose as described in Methods, followed by spheroplasting and subsequent microarray labeling and detection. Shown are the ratios of "end label" of Gal/Glu samples after Lowess smoothing or the chromosome breakage profile for Chr II, III and VI. The positions of the HO cut sites are indicated by arrows. Note that the spurious peaks on Chr III at 42, 106, 232 and 250 kb as well as the majority of spurious peaks on Chr II and VI are all due to a singleton outlier probe on the microarray. In contrast, all the chromosome breakage peaks at the HO cut sites are comprised of approximately 100 consecutive probes on the microarray.
