# Supplementary material for: Replication Stress-Induced Chromosome Breakage Is Correlated with Replication Fork Progression and Is Preceded by Single-Stranded DNA Formation
Source: G3 (Bethesda). 2011 Oct 1;1(5):327–35. doi: 10.1534/g3.111.000554 (PMC3276152; doi:10.1534/g3.111.000554)
Supplement: Supporting Information [file supp_1.5.327_FigureS5.pdf]

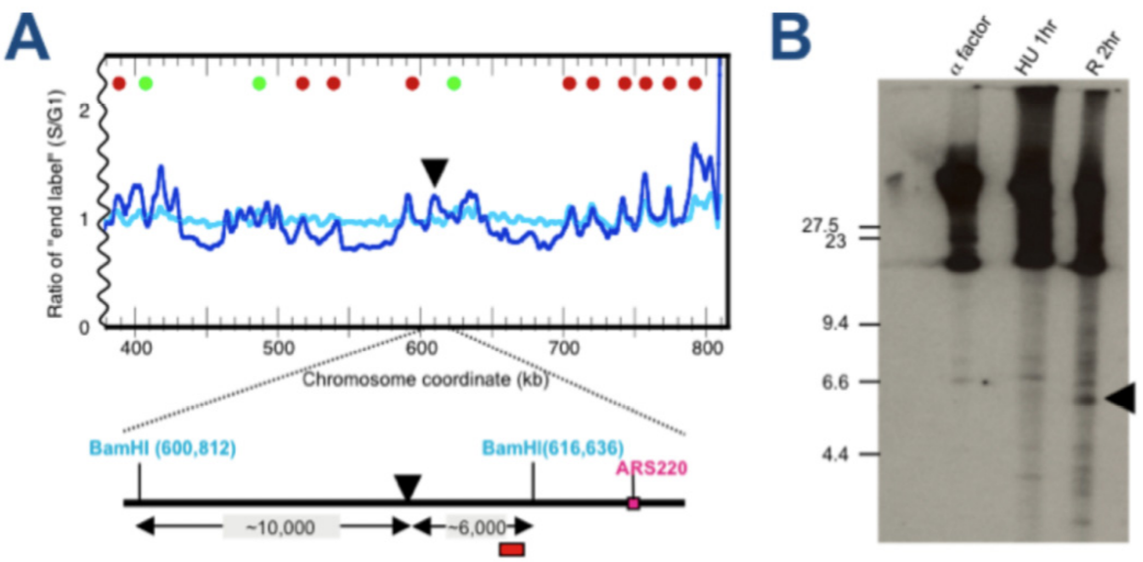

Figure S5 Verification of chromosome breakage site on Chr II by indirect end-labeling as previously described [8]. (A) Top: Chromosome breakage profile for the right half of Chr II for the HU 1hr (cyan) and the R 1hr (dark blue) samples. The breakage site chosen for analysis is indicated by an arrowhead. The Rad53-checked (red) and unchecked origins (green) are indicated as dots on top of the graph. Bottom: Structure of the *Bam*HI fragment harboring the break site that is analyzed by indirect end-labeling. The position of the Southern probe is indicated by a red bar. (B) Chromosomal DNA from *mec1* cells from the G1 control ("α factor"), after 1 hr exposure to HU ("HU 1hr") and recovering for 2 hr after HU exposure ("R 2 hr") were embedded in agarose and in-gel digested with *BamH*I before electrophoretic separation. Southern blotting was performed with the probe indicated in (A). The predicted fragment in the "R 2 hr" sample as a result of the breakage is indicated. Molecular weight markers in "kb" are shown on the left.
